# The influence of cardiorespiratory fitness level on the relationship between work rates at the aerobic threshold (AerT) and the point of maximal fat oxidation (Fat_max_) in untrained adults

**DOI:** 10.3389/fspor.2024.1321896

**Published:** 2024-02-23

**Authors:** Martin Pühringer, Susanne Ring-Dimitriou

**Affiliations:** Department of Sport and Exercise Science, University of Salzburg, Salzburg, Austria

**Keywords:** exercise testing (CPET), exercise physiology, aerobic capacity, aerobic threshold (AerT), point of maximal fat oxidation (Fat_max_), fat oxidation capacity, agreement

## Abstract

**Introduction:**

In this study, we investigated the impact of cardiorespiratory fitness (CRF), quantified as peak oxygen consumption (VO_2peak_), on the relationship between work rates (WR) at the aerobic threshold (AerT) and the point of maximal fat oxidation rate (Fat_max_).

**Methods:**

A total of 761 untrained adults aged 41–68 completed a one-minute incremental exercise test on a cycle ergometer, using breath-by-breath gas analysis to determine VO_2peak_, AerT, and Fat_max_. AerT was determined using automatic and visual detection methods, and Fat_max_ was determined using indirect calorimetry. Participants were categorized into CRF-groups: low (<25th percentile), medium (≥25th percentile and <75th percentile), and high (≥75th percentile).

**Results:**

Fat_max_ was found at 43 ± 7% WR_peak_, 37% ± 6% WR_peak_ and 35% ± 7% WR_peak_ in the low, medium, and high CRF-groups, respectively. In contrast, AerT was located at significantly higher relative work rates: 51% ± 8% WR_peak_, 47% ± 10% WR_peak_, and 47% ± 11% WR_peak_ in the respective CRF-groups. There was a weak agreement between Fat_max_ and AerT [intraclass correlation coefficient (ICC) = .19, *p* < .001], and the ICC decreased from .35 to .12 to .13, while the mean bias ±95% limits of agreement increased from 8% ± 14% WR_peak_ to 8% ± 19% WR_peak_ to 12% ± 44% WR_peak_ from CRF-low to CRF-medium to CRF-high. The mean difference between Fat_max_ and AerT was significantly different among the CRF subgroups: 8% ± 7% WR_peak_ vs. 10% ± 10% WR_peak_ vs. 12% ± 11% WR_peak_ in low, medium, and high CRF-groups, respectively. Nonetheless, multiple regression analysis revealed only a weak positive correlation between the difference in relative work rates (% WR_peak_) between Fat_max_ and AerT (dependent variable) and the predictor variables CRF and sex, both identified as significant (*R* = .19, *p* < .001).

**Conclusion:**

Our study confirms substantial differences in exercise intensities between Fat_max_ and AerT in untrained adults (10% ± 19% WR_peak_, ranging from −14% to 53% WR_peak_). Importantly, this difference remains relatively consistent across varying CRF levels, emphasizing the distinct nature of Fat_max_ and AerT, with CRF playing a limited role in influencing their relationship in our study's untrained adults.

## 1 Introduction

In recent years, exercise physiology has witnessed a surge of interest in the examination of various physiological indices for monitoring exercise performance and prescribing optimal exercise intensities for health promotion and therapy. Among these indices, the Point of Maximal Fat Oxidation (Fat_max_) and the Aerobic Threshold (AerT), have gained significant attention due to their crucial roles in optimizing exercise prescription and performance enhancement (1–4). However, the agreement and relationship between Fat_max_ and AerT remain subjects of debate, underscoring the need for further research (5, 6).

During physical activity, lipids and carbohydrates (CHO) serve as the primary energy sources in humans, and their utilization is influenced by factors such as exercise intensity, duration, fitness level, sex, time of day, and nutritional status (7–10). At low and moderate exercise intensities, absolute and relative fat oxidation rates increase until reaching Fat_max_, after which they decline with further intensity increases, eventually reaching a minimum fat oxidation rate (Fat_min_). Concurrently, CHO oxidation rates increase with exercise intensity, becoming the dominant energy source at and above Fat_min_, particularly at the heavy and severe exercise intensity domains (11, 12). High potential for fat oxidation is indicative of metabolic fitness and holds significance for exercise performance and health (12, 13).

These exercise-induced alterations in energy metabolism have been associated with changes in oxygen consumption (VO_2_) and carbon dioxide output (VCO_2_), which are measured non-invasively through breath-by-breath gas analysis during a cardiopulmonary exercise test (CPET) (2, 14, 15). As a result, a three-phase model with two submaximal indices delineating these phases, specifically the AerT and the Anaerobic Threshold (AnT), has been established (2). Over the years, various terminologies have been employed to describe these two submaximal indices, leading to confusion and misunderstandings (16). For further clarification regarding the physiological and methodological significance of these indices, we recommend referring to the following sources (2, 3, 15, 16). However, in this paper we align with the conceptual framework for performance diagnosis and training prescription proposed by Meyer et al. (2), which provides a clear description of these indices and the three-phase model.

The initial rise in blood lactate concentration during an incremental exercise test leads to a disproportionate increase in VCO_2_ relative to VO_2_. This phenomenon is attributed to excess CO_2_ generated during the bicarbonate buffering of hydrogen ions resulting from lactic acid dissociation. This distinctive gas exchange pattern serves as a hallmark used to identify the AerT employing the *v*-slope method (14, 17) and indicates the transition from primarily aerobic energy metabolism (involving primarily fatty acid oxidation and aerobic glycolysis; phase one) to a partially anaerobic energy metabolism (involving mainly aerobic and anaerobic glycolysis; phase two) (2, 3). It has been shown, that AerT is a predictor of physical performance, morbidity, and mortality, and is dependent on age, the training and health status of individuals (2–4). In professional athletes with high levels of CRF, AerT corresponds to 70%–75% VO_2peak_, whereas it occurs at considerably lower intensities in individuals with a less extensive endurance training background (e.g., at 65% VO_2peak_ in non-professional but well-trained cyclists). In physically fit or healthy sedentary adults, AerT rarely reaches or surpasses 60% VO_2peak_ (2).

Since the introduction of the Fat_max_ concept by Jeukendrup and Achten (1), researchers have explored the potential connection between exercise intensities at Fat_max_ and AerT (6). Fat_max_ has been observed to occur at or below 48% VO_2peak_ in untrained individuals (10, 18, 19), corresponding to exercise intensities below AerT (8, 10, 19). Moreover, the adaptability of Fat_max_ to training and increased habitual physical activity is well documented (18, 20–22). Conversely, intriguingly, some studies have reported that Fat_max_ and AerT align closely, with only marginal difference, in moderately trained men (45 vs. 46% VO_2peak_) (23). However, it is important to note that substantial inter-individual variations in exercise intensity at Fat_max_ exist among specific population groups (10, 11, 24). In a recent meta-analysis focusing on the agreement between AerT and Fat_max_ (5), the reported mean bias ±95% limits of agreement (LoA) between Fat_max_ and AerT was −6% ± 20% VO_2peak_, indicating a notable discrepancy between these indices.

However, there are critical gaps in the existing body of research. Most studies included in the aforementioned meta-analysis had relatively small sample sizes (ranging from *n* = 13 to *n* = 56), with only two studies involving larger cohorts exceeding *n* = 100. Furthermore, the age distribution of participants was predominantly skewed towards individuals in their thirties, resulting in a lack of data on older adults. This shortage of comprehensive studies with larger and more diverse populations, particularly in older age groups, hinders a nuanced understanding of the relationship between Fat_max_ and AerT.

Additionally, it is worth noting that methodological variations, including differences in ergometer types, test protocols, and Fat_max_ and AerT detection methods, as well as moderating factors like sex and physical activity, play pivotal roles in shaping the relationship between these indices. Notably, the influence of cardiorespiratory fitness (CRF) on this association remains an underexplored aspect in the current literature (5). Furthermore, recent discourse has raised questions about the accuracy of expressing exercise intensity as a fixed percentage of maximal values, e.g., % VO_2peak_ or % HF_peak_ (23, 25). Consequently, some researchers advocate for a more personalized approach to exercise prescription, one based on work rates at submaximal indices. This approach allows for a more precise and individualized exercise intensity prescription (25).

Building upon these insights and leveraging a comprehensive dataset, our paper seeks to expand upon the current body of knowledge by further exploring the relationship between the relative work rates corresponding to Fat_max_ and AerT. A key aspect of our research is our focus on untrained adults aged 55 ± 4 years, a demographic often underrepresented in previous studies. Additionally, we consider the potential influence of CRF level on this relationship. Through this, we strive to provide invaluable insights for the precise and tailored prescription of exercise intensities.

## 2 Methods

### 2.1 Participants

**Figure 1:**
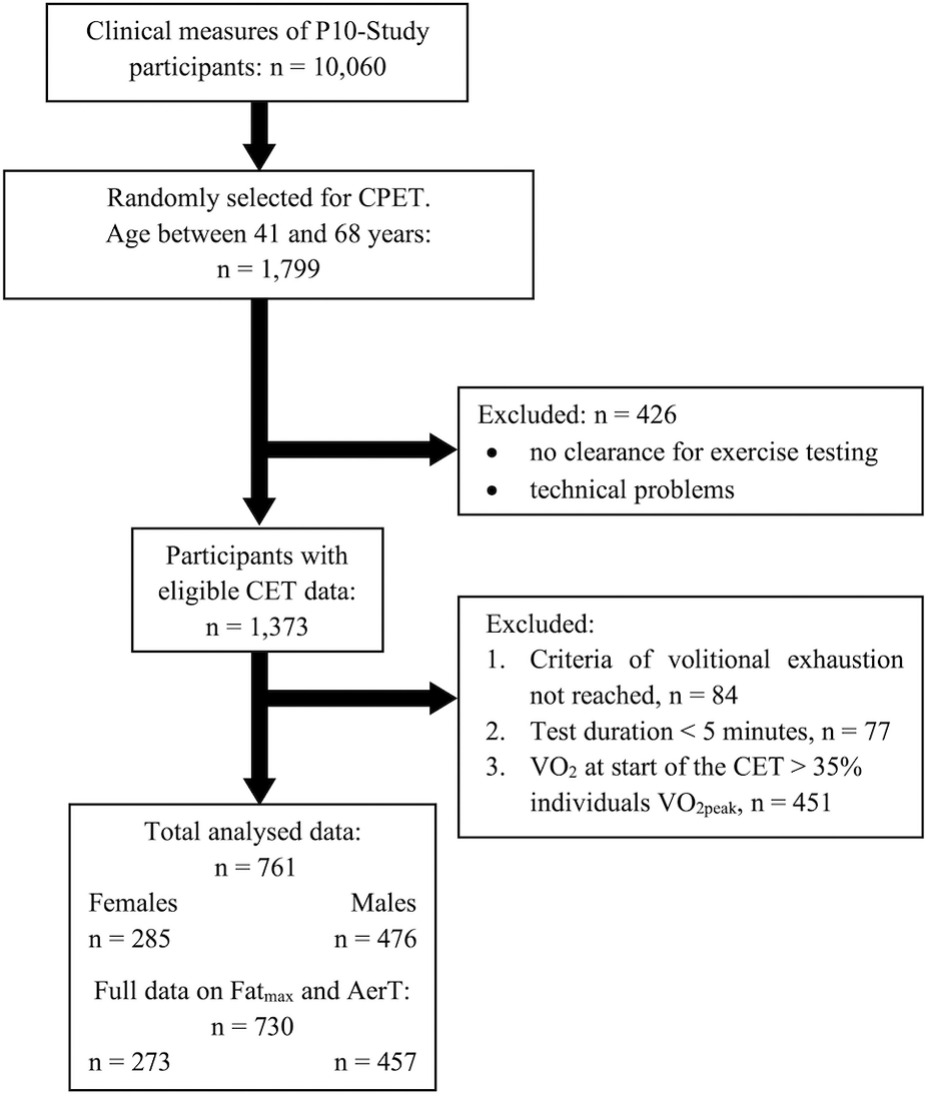
Cardiopulmonary exercise test (CPET) participant flow.

This is a cross-sectional study involving 761 datasets (284 females and 477 males). These datasets were drawn from a sub-sample of 1,372 participants who were part of the Paracelsus 10,000 Study (P10-Study) and were randomly selected to undergo CPET. The P10-Study, conducted between 2013 and 2020, is an observational study with the primary objective of assessing the health status of 10,060 randomly selected individuals aged 40 to 70 years residing in Salzburg, Austria (26). For our analysis, we selected participants who met the criteria of volitional exhaustion during CPET and who completed at least five minutes of the exercise test (see Figure 1 and Table 2). Participants were categorized into the following CRF subgroups based on the American College of Sports Medicine guidelines (27), with those below the 25th percentile classified as having low or very low CRF levels, and those above the 75th percentile classified as having good or excellent CRF levels: (1) CRF-low: Representing those below the 25th percentile, with VO_2peak_ values <22.6 ml · kg^−1^ · min^−1^ in females and <26.5 ml · kg^−1^ · min^−1^ in males. (2) CRF-medium: Representing those between the 25th and 75th percentiles, with VO_2peak_ values ≥22.6 ml · kg^−1^ · min^−1^ and <29.1 ml · kg^−1^ · min^−1^ in females, and ≥26.5 ml · kg^−1^ · min^−1^ and <35.6 ml · kg^−1^ · min^−1^ in males. (3) CRF-high: Representing those above the 75th percentile, with VO_2peak_ values ≥29.1 ml · kg^−1^ · min^−1^ in females and ≥35.6 ml · kg^−1^ · min^−1^ in males. The P10-Study adhered to the principles outlined in the Declaration of Helsinki and received approval from the regional ethics committee of the federal state of Salzburg (415-E/1521/3-2012). All participants provided written informed consent.

### 2.2 Data collection

The data collection procedures were standardized and conducted at Salzburg University Hospital, Austria, between 7:00 a.m. and 3:00 p.m. on a single day. Participants were given specific instructions to abstain from consuming coffee or smoking on the test day and were provided with standardized meals. CPET measurements were conducted between 1:00 and 3:00 p.m.

#### 2.2.1 Participant characteristics and medical examinations

Medical examinations conducted by qualified physicians included a comprehensive medical history assessment and physical examinations, anthropometric and standard spirometric measurements, laboratory evaluations (covering blood chemistry, haematology, and urine analysis), and electrocardiograms. Body fat mass (FM) and fat-free mass (FFM) were estimated by multi-frequency bio-impedance analysis (B.I.A Nutriguard-M, Data Input, Darmstadt, Germany). Therefore, electrodes (Bianostic AT, Data Input, Darmstadt, Germany) were attached on the frontal site of the left wrist and ankle of the participant in a supine position, following the manufacturer's guidelines (Data Input, Darmstadt, Germany).

#### 2.2.2 CPET and gas exchange measurements

Following the medical examination, participants underwent incremental exercise testing. Exclusion criteria for CPET included anaemia, severe cardiovascular disease, paralysis, extremity abnormalities, or any subjective limitations such as pain or musculoskeletal disorders (26). During exercise, continuous respiratory gas analysis and volume measurements were performed using a facemask (Hans Rudolph, Kansas, USA) to ensure an airtight seal over the participant's nose and mouth. This facemask was equipped with an attached volume sensor (Triple-V®) and a gas analyzer (Master Screen CPX; Accuracy: VE: 2%, VO_2_: 3%, VCO_2_: 3%), connected using a semipermeable sampling tube (Twin Tube; all products are manufactured by Jaeger, Höchberg, Germany). The following parameters were recorded breath-by-breath throughout the exercise and registered as raw data: VO_2_, VCO_2_, VE, end-tidal partial pressure of oxygen and carbon dioxide (PETO_2_, PETCO_2_), ventilatory equivalents of O_2_ and CO_2_ (EQO_2_, EQCO_2_). Equipment calibration was conducted daily by medical technicians, following the instruction manual, using the inbuilt calibration tools and a reference gas mixture (mixture of 5% CO_2_, 16% O_2_, 79% N_2_; Rießner Gase GmbH, Lichtenfels, Germany).

### 2.3 CPET and exercise protocol

Participants in this study underwent an incremental exercise test until reaching the point of volitional exhaustion. The exercise protocols aimed to induce exhaustion within a duration of 8–12 min (as outlined in Table 1) using individualized starting workloads and increments, taking into account factors such as sex and body mass, following established guidelines (4, 27).

**Table 1:** Stationary cycling protocols of the P10-study for CPET.

	Females			Males			Females and males
Body mass range, kg	50–69	50–69	70–94	50–69	70–94	70–94	95–119
Initial workload, *W*	40	50	60	50	70	70	90
Increment, *W* · min^−1^	10	10, 15^a^	10, 15^a^	15	15, 20^a^	20	20, 25^a^

^a^ Increment rise after 6th minute to ensure a test duration of about 10–12 min.

The exercise tests were conducted on a cycle ergometer (ergo select 200P, ergo line GmbH, Bitz, Germany), with seat height and handlebar positions adjusted individually for each participant. The testing procedure commended with a two-minute stationary phase without pedaling, allowing participants to accustomed to breathing through the mask. This was followed by a two-minute warm-up period at 10 W. Subsequently, participants engaged in an incremental exercise test, increasing the workload every minute until reaching volitional exhaustion, while maintaining a pedaling rate of 60 rpm. After exhaustion, a five-minute recovery phase at 10 W was administered.

Confirmation of volitional exhaustion, and thereby the attainment of VO_2peak_, was based on meeting at least two of the following criteria (14):

• A plateau in VO_2_ (indicating minimal changes of less than 2 ml · kg^−1^ · min^−1^ following an increase in workload)
• EQO_2_ > 30
• Respiratory exchange ratio (RER)  > 1.1
• Achievement of 90% of the age-predicted maximum heart rate (28)
• pedaling rate falling below 50 rpm due to leg fatigue or shortness of breath

Exercise testing was terminated if any complications or contraindications occurred (4).

### 2.4 Data processing

Data from the stationary cycling test (CPET) regarding the warm-up and recovery phase were excluded from further analyses. The recorded breath-by-breath data were averaged over 10-s epochs and then aligned at the top of each 10-s epoch. The mean of the three consecutive highest 10-s VO_2_ values at cessation was then taken as the peak value. Peak work rate (WR_peak_) was determined as the mean work rate during the last minute of the exercise test (29, 30).

Fat_max_ is typically found at low to moderate exercise intensities and was reported at 38%–64% VO_2peak_ (11, 21), respectively. Therefore, participants with a VO_2_ of more than 35% of their individual VO_2peak_ at the onset of stationary cycling were excluded from further analysis because the initially applied work rate might have been too high in these individuals to accurately determine Fat_max_. Additionally, participants who could not complete at least five minutes of the CPET were excluded. These two exclusion criteria were implemented to ensure the detectability of Fat_max_ and AerT, as well as to guarantee that exhaustion would be reached.

#### 2.4.1 Determination of Fat_max_ and AerT

The VO_2_ and VCO_2_ 10-s averages were used to calculate fat oxidation rates according to the non-protein respiratory exchange ratio (RER) technique, assuming negligible urinary nitrogen excretion rates (11). For each participant, the calculated values for fat oxidation were graphically depicted as a function of exercise intensity (% VO_2peak_), and a 3rd degree polynomial function with an intersection at point zero was constructed to determine the relative intensity that elicited the highest rate of fat oxidation (Fat_max_) (11, 31). If fewer than six calculated fat oxidation values where available to construct the third-degree polynomial, the data was excluded from further analysis (32).

The ventilatory index AerT was determined semi-automatically by combining automatic and visual detection methods (33). Initially, the maximum curvature in the VCO_2_ vs. VO_2_ plot was calculated. Subsequently, the time point of AerT during the exercise test was visually determined by identifying the first disproportional increase in the VCO_2_ vs. VO_2_ plot (17), with the calculated AerT indicated on the plot for guidance. Additional guidance and verification of the selected AerT from the first step were provided through EQCO_2_, PETCO_2_, EQO_2_, and PETO_2_ time plots. Finally, the selected time point was used to determine the work rate and VO_2_ at AerT (33).

### 2.5 Statistical analysis

Data are presented as means ± standard deviations and frequencies. Differences in participant characteristics, resting spirometry, and CPET variables between sexs and CRF-groups were assessed using a two-way ANOVA (sex, CRF-groups) with Bonferroni-adjusted *post-hoc* testing. The level of absolute agreement between relative work rate at Fat_max_ and AerT was evaluated for the total sample as well as separately for females and males, and individual CRF-groups by calculating intraclass correlation coefficients (ICC [95% confidence interval) based on a single-rater, absolute-agreement, and a two-way mixed-effects model (34). Additionally, Pearson's product-moment correlation (*r*) and mean biases ±95% LoA according to (35) were calculated.

A two-way ANOVA [CRF-group, indices (Fat_max_ and AerT)] with Bonferroni-adjusted *post-hoc* testing was used to analyze differences in relative work rates between Fat_max_ and AerT and between CRF-groups. Additionally, multiple regression analysis was used to predict the difference in relative work rates at Fat_max_ and AerT, incorporating CRF level (expressed as VO_2peak_), sex, age and BMI as predictor variables. To visually depict the strength of the linear relationships between the dependent variable and each single predictor variable, added variable plots were generated.

One-way ANOVA (CRF-group) with Bonferroni-adjusted *post-hoc* testing was conducted to evaluate differences between CRF-groups in the relative work rate difference between Fat_max_ and AerT. The level of significance was set at *α* < .05. Statistical analyses were performed using RStudio version 2023.06.2 + 561 (RStudio Inc., Boston, Massachusetts, USA).

## 3 Results

### 3.1 Participant characteristics

Participant characteristics of the 284 females and 477 males who successfully completed the CPET and met the inclusion criteria for this study are summarized in Table 2. In comparison to reference values published by Rapp et al. (36), our female and male participants exhibited cardiorespiratory fitness levels approximately corresponding to the 15th, 45th and 85th percentiles (expressed as VO_2peak_) in the CRF-low, CRF-medium, and CRF-high group, respectively. Pulmonary function at rest, as indicated by average FVC and FEV1 values, was within the normal range in both females (3.6 ± 0.6 L and 2.7 ± 0.4 L, respectively) and males (4.9 ± 0.7 L and 3.6 ± 0.6 L, respectively) (37).

**Table 2:** Characteristics, comorbidity and main results of resting spirometry and cardiopulmonary exercise test (CPET) for the three cardiorespiratory fitness (CRF) groups, separately for females and males.

	Females						Males					
CRF subgroup	Low		Medium		High		Low		Medium		High	
*n*	71		142		71		119		238		120	
	M	SD	M	SD	M	SD	M	SD	M	SD	M	SD
Characteristics												
Age, years	54	4	54	3	54	3	56	4	55	4	55	4
Height, cm	166	5	166	6	166	5	179	7^†^	179	6^†^	178	6^†^
Body mass, kg	74	13*	66	10^‡^	60	6^‡^^,^^*^	90	12^*^^,^^†^	84	11^‡^^,^^†^	76	8^‡^^,^^*^^,^^†^
FFM, kg	47	5	46	5	45	4	67	7^*^^,^^†^	65	8^†^	62	6^‡^^,^^*^^,^^†^
FM, kg	26	9*	21	7^‡^	15	4^‡^^,^^*^	23	7^*^^,^^†^	19	6	14	5^‡^^,^^*^
BMI, kg · m^2^	27	4*	24	3^‡^	22	2^‡^^,^^*^	28	3*	27	3^‡^^,^^†^	24	2^‡^^,^^*^^,^^†^
Waist circumference, cm	91	11*	84	9^‡^	79	7^‡^^,^^*^	102	10^*^^,^^†^	97	8^‡^^,^^†^	89	6^‡^^,^^*^^,^^†^
Resting Spirometry												
FVC, *L*	3.6	0.7	3.6	0.5	3.7	0.4	4.6	0.7^*^^,^^†^	4.9	0.7^‡^^,^^†^	5.0	0.7^‡^^,^^†^
FEV_1_, *L*	2.6	0.5	2.7	0.4	2.8	0.3	3.5	0.6^*^^,^^†^	3.6	0.5^†^	3.8	0.5^‡^^,^^†^
CPET												
VO_2_ at Fat_max_, ml ·kg^−1^· min^−1^	8.6	2.1*	10.5	2.1^‡^	13.1	2.9^‡^^,^^*^	10.4	1.8^*^^,^^†^	13.2	2.3^‡^^,^^†^	16.9	3.3^‡^^,^^*^^,^^†^
VO_2_ at AerT, ml ·kg^−1^· min^−1^	10.5	2.3*	13.9	2.6^‡^	17.3	3.7^‡^^,^^*^	12.7	2.1^*^^,^^†^	16.3	2.9^‡^^,^^†^	21.2	4.7^‡^^,^^*^^,^^†^
VO_2peak_, ml ·kg^−1^· min^−1^	19.7	2.2*	25.6	1.9^‡^	33.2	3.2^‡^^,^^*^	23.7	2.0^*^^,^^†^	30.5	2.5^‡^^,^^†^	39.4	3.8^‡^^,^^*^^,^^†^
%VO_2peak_ at Fat_max_, %	44	9	41	7	39	8^‡^	44	7	43	7	43	7^†^
%VO_2peak_ at AerT, %	53	9	54	10	52	9	53	8	54	9	54	10
%WR_peak_ at Fat_max_, %	43	7	36	6^‡^	34	7^‡^	42	6	37	6^‡^	36	7^‡^
%WR_peak_ at AerT, %	52	8	48	10	47	11^‡^	50	8	47	9^‡^	48	11
WR_peak_, W · kg^−1^	1.9	0.3*	2.3	0.3^‡^	2.9	0.3^‡^^,^^*^	2.2	0.3^*^^,^^†^	2.8	0.3^‡^^,^^†^	3.6	0.4^‡^^,^^*^^,^^†^
HR_peak_, min^−1^	160	13	164	12	167	11^‡^	157	14*	164	13^‡^	167	12^‡^
RER_peak_	1.17	0.08	1.20	0.07	1.18	0.07	1.21	0.07^†^	1.21	0.08	1.18	0.07^‡^^,^^*^
Comorbidity	*n* (%)		*n* (%)		*n* (%)		*n* (%)		*n* (%)		*n* (%)	
Hypertension	7 (10)		11 (8)		3 (4)		31 (26)		35 (15)		10 (8)	
Pulmonary disease	6 (9)		11 (8)		2 (3)		14 (12)		21 (9)		9 (8)	
Diabetes mellitus	0 (0)		2 (1)		0 (0)		7 (6)		6 (3)		1 (1)	
Cardiovascular disease	3 (4)		7 (5)		2 (3)		9 (8)		19 (8)		9 (8)	

Data are presented as means (M) ± standard deviations (SD) or numbers (*n*) and frequencies (%): FFM, fat-free mass; FM, fat mass; BMI, body mass index; FVC, forced vital capacity; FEV_1_, forced expiratory volume over 1 s; Fat_max_, point of maximal fat oxidation; AerT, aerobic threshold; VO_2_, oxygen uptake; WR, work rate; HR, heart rate; RER, respiratory exchange ratio.

* *p* < .05 vs. CRF-medium.

^†^ *p* < .05 vs. females.

^‡^ *p* < .05 vs. CRF-low.

Notably, female participants in this study were significantly smaller, lighter and had a lower waist circumference compared to their male counterparts. Additionally, sex differences in VO_2peak_ and WR_peak_ were detected. Furthermore, significant differences were found between the CRF subgroups in body mass, FM, FFM and BMI. Specifically, the CRF-low group was significantly heavier with higher BMI and FM compared to the CRF-high groups in both males and females. Additionally, relative work rate (expressed as % WR_peak_) and relative VO_2_ (expressed as % VO_2peak_) at Fat_max_ showed a significant decrease from the CRF-low to the CRF-high group in females, while only a significant decrease in relative work rate was observed in males. Furthermore, relative VO_2_ at Fat_max_ was significantly higher in males within the CRF-high group compared to their female counterparts. No differences were detected in relative VO_2_ at AerT between CRF groups or between males and females. However, the relative work rate at AerT exhibited significant differences between CRF-high and CRF-low groups in females, as well as between CRF-medium and CRF-low groups in males.

### 3.2 Differences and agreement between relative work rates at Fat_max_ and AerT

**Figure 2:**
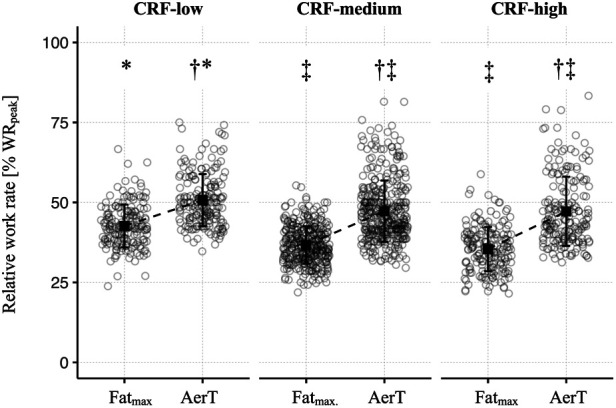
Means (squares) ± standard deviations (lines) of the relative work rate (% WR_peak_) at Fat_max_ and AerT, shown separately for CRF-groups. The circles refer to individual values at the indices Fat_max_ and AerT, respectively. Additionally, adjusted *p*-values of *post-hoc* comparisons between indices (Fat_max_ and AerT) and CRF-groups are displayed: ^†^*p* < .05 vs. Fat_max_; ^‡^*p* < .05 vs. CRF-low; *p < .05 vs. CRF-medium.

A two-way ANOVA revealed a statistical significant difference between relative work rates (% WR_peak_) at Fat_max_ and AerT [*F* (1, 727) = 714; *p* < .001, *η*^2^*_p_* = .50] and between CRF-groups [*F* (2, 727) = 38; *p* < .001, *η*^2^*_p_* = .10]. A statistically significant interaction between the two indices Fat_max_ and AerT and CRF-group was also observed [*F* (2, 727) = 8; *p* < .001, *η*^2^*_p_* = .02]. *Post-hoc* analysis revealed significant differences between Fat_max_ and AerT in all three CRF subgroups, indicating, that AerT is found at a significantly higher relative work rate than Fat_max_ (see Figure 2 and Table 3).

**Table 3:** Means ± standard deviations of relative work rate (% WR_peak_) at Fat_max_ and AerT. Intraclass Correlation Coefficients (ICC), Pearson's product-moment correlation coefficient (*r*) and mean bias ± 95% limits of agreement (LoA) for comparisons between Fat_max_ and AerT. Minimum (min) and maximum (max) range for differences between relative work rates at Fat_max_ and AerT.

				Comparisons			
		Fat_max_ [% WR_peak_]	AerT [% WR_peak_]	ICC [95% CI]	*r*	Mean bias ±95% LoA [% WR_peak_]	Range (min; max) [% WR_peak_]
Total	Total, *n* = 730	38 ± 7	48 ± 10	.19 [-.05; .39]	.35	10 ± 19	−14; 53
	CRF-low, *n* = 179	43 ± 7	51 ± 8	.35 [-.06; .62]	.55	8 ± 14	−7; 32
	CRF-medium, *n* = 364	37 ± 6	47 ± 10	.12 [-.05; .28]	.26	8 ± 19	−11; 53
	CRF-high, *n* = 187	35 ± 7	47 ± 11	.13 [-.05; .30]	.26	12 ± 22	−14; 53
Females	Total, *n* = 273	37 ± 8	49 ± 10	.17 [-.06; .37]	.32	11 ± 20	−11; 53
	CRF-low, *n* = 68	43 ± 7	52 ± 8	.34 [-.07; .62]	.53	8 ± 14	−7; 27
	CRF-medium, *n* = 137	36 ± 6	48 ± 10	.09 [-.06; .24]	.20	12 ± 21	−11; 53
	CRF-high, *n* = 68	34 ± 7	47 ± 11	.10 [-.07; .29]	.21	12 ± 23	−5; 45
Males	Total, *n* = 457	38 ± 7	48 ± 10	.21 [-.04; .42]	.38	8 ± 18	−14; 53
	CRF-low, *n* = 111	42 ± 6	50 ± 8	.36 [-.06; .63]	.57	8 ± 13	0; 32
	CRF-medium, *n* = 227	37 ± 7	47 ± 9	.16 [-.05; .35]	.31	9 ± 18	−7; 40
	CRF-high, *n* = 119	36 ± 7	48 ± 11	.14 [-.06; .33]	.29	11 ± 22	−14; 53

**Figure 3:**
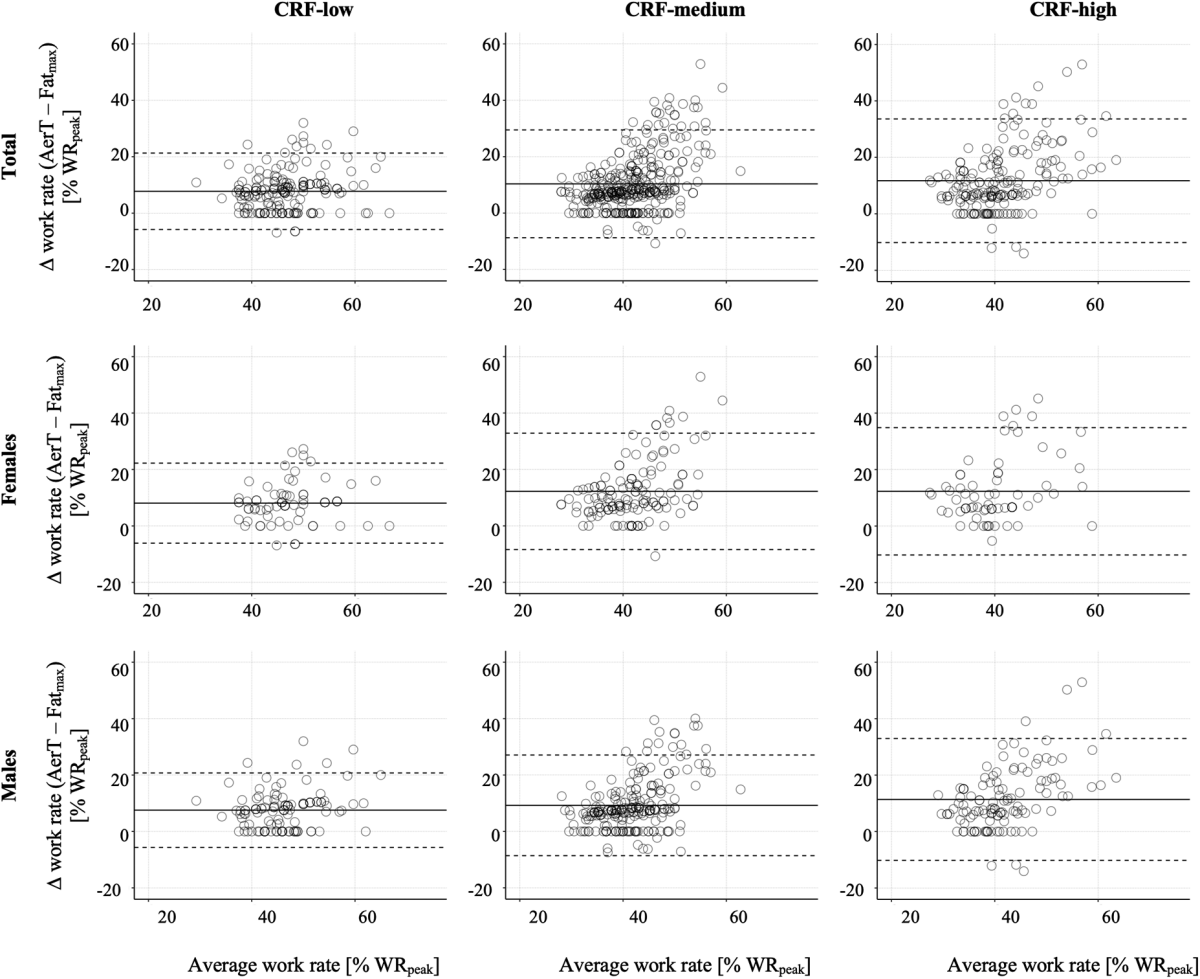
Bland–Altman plots of the differences in relative work rate (% WR_peak_) between Fat_max_ and AerT vs. the mean of their values, shown separately for CRF-groups. The solid horizontal line represents the mean bias between the two indices (Fat_max_ and AerT), and the top and bottom dashed lines represent the 95% limits of agreement [1.96 · standard deviation].

We employed a Bland-Altman limit of agreement analysis to investigate the absolute agreement between the submaximal indices Fat_max_ and AerT. The mean bias was 10% WR_peak_ in the total sample and increased slightly from the CRF-low (8% WR_peak_) to the CRF-high (12% WR_peak_) group. Accordingly, the 95% LoA also increased substantially from 14% WR_peak_ in the CRF-low to 22% WR_peak_ CRF-high group. Furthermore, separate Bland-Altman analysis were conducted for females and males, revealing similar patterns with only marginal differences between females and males compared to the total sample, as described above for mean bias ±95% LoA (Table 3 and Figure 3).

**Figure 4:**
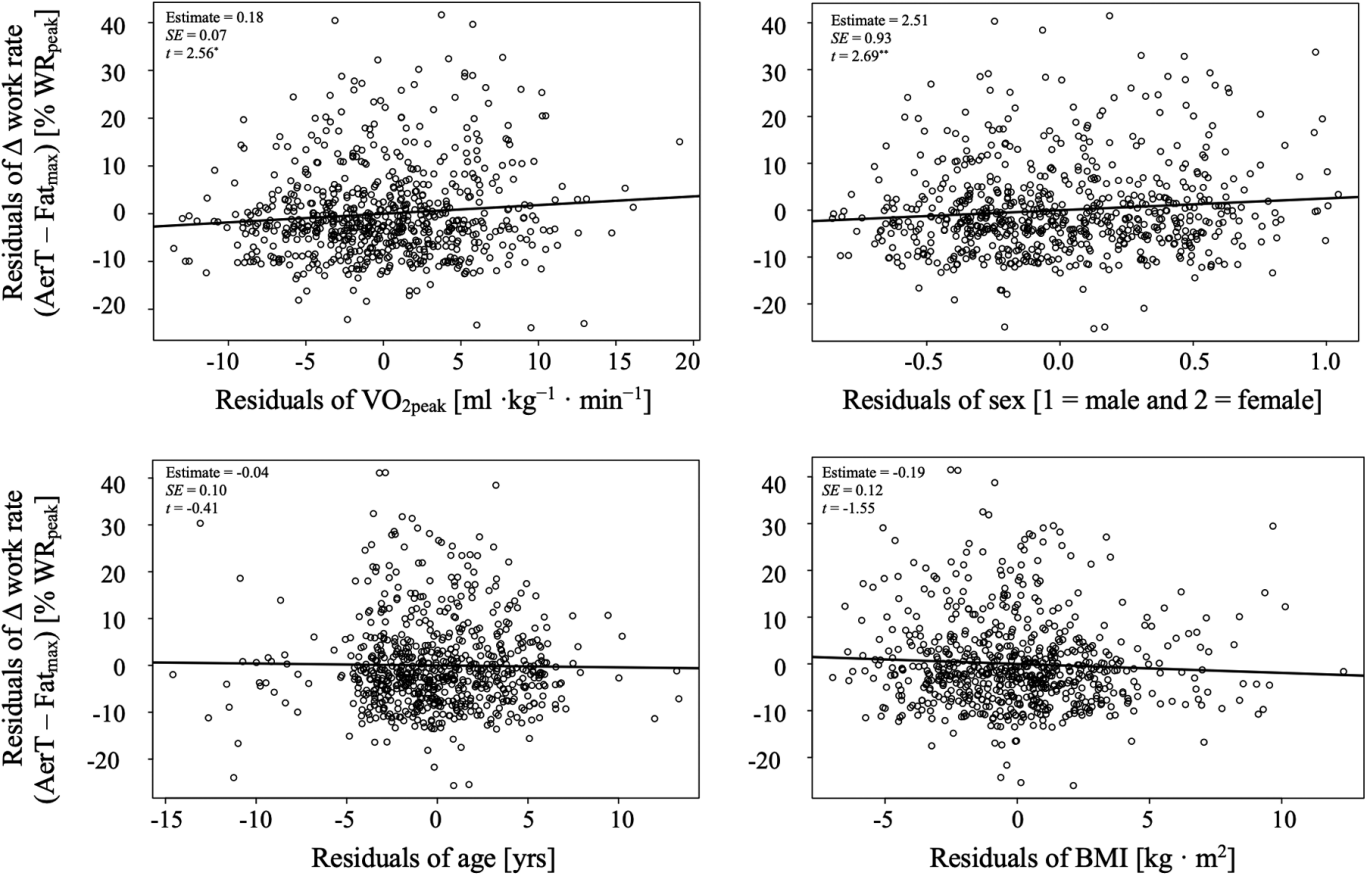
Added variable plots from multiple regression model in table 4.

The mean (± standard deviation) difference between Fat_max_ and AerT was significantly different between the CRF subgroups: 8% ± 7% WR_peak_ vs. 10% ± 10% WR_peak_ vs. 12% ± 11% WR_peak_ in the CRF-low vs. CRF-medium vs. CRF-high group, respectively [*F* (2, 727) = 8; *p* < .001, *η*^2^*_p_* = .02]. *Post-hoc* analysis revealed significant difference between CRF-low and CRF-medium (*p* < .010) as well as between CRF-low and CRF-high (*p* < .001). Multiple regression analysis was conducted with the difference in relative work rate at Fat_max_ and AerT as the dependent variable. The predictor variables included CRF level (expressed as VO_2peak_), sex, age, and BMI. The results indicate that only CRF level and sex are significant predictors of the difference in relative work rate at Fat_max_ and AerT. However, the overall model's *R* was.19 [*F* (4, 687) = 6.26, *p* < .001], and the *R*^2^_adj_ was.03, suggesting only a weak positive correlation (see Table 4 and Figure 4).

**Table 4:** Multiple linear regression analysis for difference in relative work rate at fat_max_ and aerT.

Dependent variable	Independent Variable	*R* ^2^	*R* ^2^_adj._	Estimate	*SE*	*t*-value
Difference in relative work rate at Fat_max_ and AerT	Intercept	.04	.03	8.59	8.23	1.04
	VO2peak			0.18	0.07	2.56*
	Sex^a^			2.51	0.93	2.69**
	Age			−0.04	0.10	−0.41
	BMI			−0.19	0.12	−1.55

^a^ 1 = male and 2 = female.

* *p* < .05.

** *p* < .01.

## 4 Discussion

In recent years, there has been a growing interest in tailoring exercise intensity prescriptions based on submaximal indices derived from CPET. Notably, two such indices, Fat_max_ and AerT, which are linked to fat oxidation capacity, have undergone intensive study. Various factors, including physical activity level, ergometer type, CPET protocol, and indices detection methods, have been identified as influencing the relationship between these indices. Nevertheless, there remains a notable debate regarding the agreement and association between Fat_max_ and AerT, highlighting the need for further investigation (5).

To address this gap, our study aimed to expand upon these findings. We explored the relationship between work rates at Fat_max_ and AerT using a comprehensive dataset comprising 761 untrained adults aged 41–68 years, a demographic often underrepresented in previous research. Additionally, we examined the potential influence of CRF levels on the agreement between Fat_max_ and AerT.

### 4.1 Participant characteristics

The results obtained from CPET, including WR_peak_ and VO_2peak_, as well as measurements of body mass, fat mass, body mass index, and waist circumference, revealed significant differences among the CRF subgroups for both females and males (as shown in Table 2). When compared to the reference values for VO_2peak_ published by Rapp et al. (36), the low, medium, and high CRF-groups presented mean VO_2peak_ levels roughly corresponding to the 15th, 45th and 85th percentiles, respectively. Despite the anticipated variations in WR_peak_ and VO_2peak_ across these subgroups, no significant differences were observed in AerT, expressed as a percentage of VO_2peak_. On average, AerT was found to occur at 53% ± 9% VO_2peak_, with a range spanning from 32% to 88% VO_2peak_. These findings align with previous studies, indicating that AerT typically does not exceed 60% VO_2peak_ in physically fit and healthy sedentary adults, as reported by Meyer et al. (2).

In contrast, Fat_max_ was consistently found at lower exercise intensities, specifically at 43% ± 8% VO_2peak_, with a range spanning from 11% to 68% VO_2peak_. These results are consistent with the values reported by Venables et al. (10), where Fat_max_ was reported at 48% ± 1% VO_2peak_, with a range from 25% to 77% VO_2peak_. Furthermore, Jeukendrup and Wallis (11) noted Fat_max_ values within the range of 33%–65% VO_2peak_, underlining that these values can be influenced by exercise mode, diet, sex, and training status. However, it's important to consider the varying stage durations and methods used during the incremental exercise tests for Fat_max_ determination across the different studies when interpreting the results mentioned above.

There was no significant difference in Fat_max_, expressed as % VO_2peak_, between males and females in the CRF-low group. However, females of the CRF-high group exhibited a significantly lower Fat_max_ compared to those in the CRF-low group. This finding may seem surprising, as Fat_max_ typically increases with an increase in VO_2peak_, as noted by Jeukendrup and Wallis (11). Nonetheless, the observed difference was relatively small (44% ± 9% vs. 39% ± 8% VO_2peak_ in CRF-low vs. CRF-high groups), and it falls within the clinically relevant Fat_max_-zone (were fat oxidation remains higher than 90% of Fat_max_) of ±9% VO_2peak_, as calculated by Ferri Marini et al. (5). Moreover, this difference can be attributed, at least in part, to the considerable inter-individual variations and the substantial variance, particularly among females in the CRF-low group (see Table 2).

### 4.2 Differences and agreement between relative work rates at Fat_max_ and AerT

In the present study, our aim was to compare relative work rates expressed as % WR_peak_ at Fat_max_ and AerT to analyze whether exercise intensity prescription would differ when based on Fat_max_ or AerT. Relative work rates at Fat_max_ were observed at 38% ± 7% WR_peak_ (38% ± 7% WR_peak_ and 37% ± 8% WR_peak_ in females and males, respectively), which were lower compared to AerT observed at 48% ± 10% WR_peak_ (see Table 2 and Table 3). These findings align with previous research (8, 10, 19) and confirm that fat oxidation is highest at low and moderate exercise intensities in untrained adults (11). However, due to the pivotal influence of test methodology on Fat_max_ estimation (e.g., ergometer type, test protocol, indices detection methods), direct comparisons with other studies should be approached cautiously (5, 11).

These findings are further supported by a weak agreement between Fat_max_ and AerT (ICC = .19, *p* < .001) and a mean bias ±95% LoA of 10% ± 19% WR_peak_ (see Table 3). This confirms previous findings (5) and suggests that practitioners may encounter differing and unintended physiological adaptations at the individual level when attempting to interchangeably use work rates from Fat_max_ and AerT for exercise intensity prescription. This is especially notable when considering the high inter-individual variation in differences between Fat_max_ and AerT, ranging from a minimum of −14% WR_peak_ to a maximum of 53% WR_peak_ in this study. Although information on inter-individual variation in relative work rates at Fat_max_ and AerT from other studies is lacking, the high variation found in our study appears reasonable when compared to the inter-individual variation in relative VO_2_ (expressed as % VO_2peak_) at Fat_max_ and AerT reported by others. Venables et al. (10) reported inter-individual variations in Fat_max_ ranging from 25% to 77% VO_2peak_, indicating the substantial inter-individual variability of Fat_max_. Additionally, in a recent meta-analysis by Ferri Marini et al. (5), pooled 95% LoA between Fat_max_ and AerT ranged from −26.5% to 13.7% VO_2peak_ (−27.7% to 14.0% VO_2peak_ for “Ergometer” subgroup: “Cycle”; and −27.7% to 12.2% VO_2peak_ for “Fat_max_ method” subgroup: “Mathematical”). Therefore, we conclude that work rates at Fat_max_ and AerT differ considerably, particularly at the individual level.

### 4.3 Influence of CRF on Fat_max_ and AerT relationship

The second aim of our study was to investigate how CRF impacts the relationship between Fat_max_ and AerT. We found significant differences in relative work rates between the low, medium and high CRF-groups in the total sample for both Fat_max_ as well as for AerT, as illustrated in Figure 2. Specifically, the relative work rate at Fat_max_ decreased from 43% ± 7% WR_peak_ in the CRF-low group to 35% ± 7% WR_peak_ in the CRF-high group. Interestingly, the decrease in AerT was less pronounced, moving from 51% ± 8% WR_peak_ to 47% ± 11% WR_peak_. Consequently, the mean bias and the 95% LoA between Fat_max_ and AerT increased from 8% ± 14% WR_peak_ in the CRF-low group to 12% ± 22% WR_peak_ in the CRF-high group, as depicted in Figure 3 and Table 3. This increase primarily results from the more substantial decrease in relative work rates at Fat_max_ from the CRF-low to the CRF-high group compared to the decline in AerT. Importantly, when we conducted a separate analysis for males and females, we observed that the trends described above remained consistent across both sexes.

Conventionally, one would anticipate an increase in Fat_max_ with improving CRF (11). Moreover, prior research has indicated that Fat_max_ tends to occur at a lower %VO_2peak_, and presumably a lower %WR_peak_, in overweight or obese individuals compared to their lean and recreationally active counterparts (21). However, our study revealed that participants in the CRF-low group had a BMI of 27 ± 4 and 28 ± 3 kg · m^−2^ in females and males, respectively, indicating overweight status, while those in the CRF-high group were characterized as lean but displayed lower Fat_max_. Therefore, we analyzed the influence of BMI and other predictors like CRF level (expressed as VO_2peak_), sex, and age on work rate differences between Fat_max_ and AerT using multiple regression analysis. However, only CRF level and sex were significant predictors of the difference in relative work rate at Fat_max_ and AerT. BMI and age did not present as significant predictors in the model.

It is important to note that our study population predominantly consisted of individuals with similar low fitness levels (i.e., untrained adults), resulting in a narrow range of potential exercise intensities for Fat_max_. Additionally, the utilization of %WR_peak_ may result in greater normalization since every individual would inevitably reach 100% WR_peak_, regardless of their absolute maximal achievable work rate. Although we made efforts to guarantee volitional exhaustion, thus reaching the absolute maximal achievable work rate, by adhering to established criteria for volitional exhaustion (14), this aspect should be kept in mind when interpreting the findings of our study.

Finally, the weak positive correlation between the significant predictors from the multiple regression analysis CRF level (expressed as VO_2peak_) and sex, and the difference in relative work rates between Fat_max_ and AerT (*R* = .19, *p* < .001) suggests only a marginal relationship.

It is noteworthy that, as indicated by Venables et al. (10), factors such as lean body mass, fat mass, physical activity level, CRF level (expressed as VO_2peak_), and sex collectively account for only 34% of the variance in peak fat oxidation rates. This implies that a significant portion of the variance remains unexplained, possibly due to influences from nutritional or genetic factors. This unexplained variance may extend to the relationship between Fat_max_ and AerT. Consequently, it appears that CRF may not serve as a crucial moderator in determining the relationship between Fat_max_ and AerT, especially in the case of untrained adults investigated in this study.

### 4.4 Limitations

There are some limitations that need to be considered for a proper interpretation of the findings reported in this study. Determining the optimal test stage duration for assessing submaximal (Fat_max_, AerT) and peak indices (VO_2peak_) in a single CPET poses a challenge. While a one-minute protocol may slightly overestimate maximal fat oxidation rate, it does not affect Fat_max_ intensity (in terms of %VO_2peak_) (32). Concerning Fat_max_ determination, other analyses approaches than the here used 3rd degree polynomial method such as the sine model method, or the measured values data method have been documented, with similar inter-individual variability between the three methods. Further, there is no basis for making a sound decision on choosing a representative point during a 1-minute stage for Fat_max_ determination using the 3rd degree polynomial method. However, using the 3rd degree polynomial function averages the data and therefore providing a reliable estimate for the intensity at Fat_max_. Amaro-Gahete et al. (38) demonstrated that there is no difference in Fat_max_ when analyzing different time intervals (e.g., first 60 s vs. last 60 s when using 3 min stages). Additionally, there is a delay in VO_2_ response to increasing exercise intensity, known as mean response time, which varies with exercise intensity and work rate increments per minute (39). Suitable stage durations may also differ among individuals based on their fitness levels. Nevertheless, shorter test protocols have been successfully used to estimate various submaximal indices in a single CPET (5, 23, 32). Furthermore, in this study, the two submaximal indices Fat_max_ and AerT are determined using analysis procedures based on the same data, specifically VO_2_ and VCO_2_ values. Therefore, one can assume that factors influencing these values will have a similar effect on both submaximal indices. In our study, we employed various one-minute stage exercise protocols and increased the increment in some protocols after the 6th minute (see Table 1) to achieve two goals: (1) facilitate a gradual work rate increase during the early stages to minimize VO_2_ response time and (2) ensure reliable VO_2peak_ values within the recommended test duration of 8 to 12 min (27). However, when comparing our results with those of other studies, it is crucial to consider differences in data collection and analytical approaches. Doing so ensures that comparisons are both meaningful and valid, avoiding ambiguities and unacceptable comparisons.

Furthermore, while participants adhered to overnight fasting and received standardized food on the test day, we did not control for their chronic nutritional status or the menstrual cycles of the female participants. It has been reported that these factors can influence both Fat_max_ and AerT (10, 40). Therefore, these factors should be taken into account in future studies on the agreement between Fat_max_ and AerT and when interpreting our results.

Lastly, the observed differences in relative work rates between Fat_max_ and AerT may, in part, be attributed to measurement errors in determining the individual indices. Addressing this issue has been a recent focus in studies by Ferri Marini et al. (5) and Peric et al. (6). Consequently, there is a need for establishing methodological standards in this regard.

## 5 Conclusion

In conclusion, our study delved into the intricate relationship between Fat_max_ and AerT, two submaximal indices with implications for tailoring exercise intensity prescription. We aimed to address gaps in the existing literature by investing these indices in a demographic often underrepresented in previous research—untrained adults aged 55 ± 4 years.

Firstly, we observed a consistent pattern where Fat_max_ consistently occurred at lower exercise intensities compared to AerT, aligning with previous research. This distinction holds significant implications for exercise practitioners, as it implies that interchangeably using one index with the other may result in unintended physiological adaptations at the individual level. The weak correlation and substantial inter-individual variation between Fat_max_ and AerT reinforce the importance of cautious application when prescribing exercise.

Secondly, we explored the influence of CRF on the relationship between Fat_max_ and AerT. Surprisingly, our findings revealed that CRF did not serve as a decisive moderator in determining this relationship among the untrained adults in our study. It is worth noting that our study primarily included individuals with similar low aerobic capacity (in terms of %VO_2peak_ at AerT). This homogeneity in aerobic capacity resulted in a relatively narrow range of potential work rates for Fat_max_ and AerT, which may account for the absence of a significant relationship. Nonetheless, this outcome underscores the need for further investigation into the multifaceted factors influencing these indices.

In summary, our study provides valuable insights into the complexities surrounding the relationship between Fat_max_ and AerT and emphasizes the importance of tailored exercise intensity prescription. While more research is needed to unravel the intricate factors at play, our findings underscore the significance of individualized exercise prescription based on a comprehensive understanding of these submaximal indices.

## Data Availability

The raw data supporting the conclusions of this article will be made available by the authors, without undue reservation.
